# A Dichoptic Optokinetic Nystagmus Paradigm for Interocular Suppression Quantification in Intermittent Exotropia

**DOI:** 10.3389/fnins.2021.772341

**Published:** 2021-12-03

**Authors:** Xiaoxiao Cai, Zidong Chen, Yanping Liu, Daming Deng, Minbin Yu

**Affiliations:** ^1^State Key Laboratory of Ophthalmology, Zhongshan Ophthalmic Center, Sun Yat-sen University, Guangzhou, China; ^2^Guangdong Provincial Key Laboratory of Social Cognitive Neuroscience and Mental Health, Department of Psychology, Sun Yat-sen University, Guangzhou, China

**Keywords:** intermittent exotropia, eye tracker, visual suppression, optokinetic nystagmus, strabismus

## Abstract

**Purposes:** To investigate the effectiveness of a dichoptic optokinetic nystagmus (dOKN) test to objectively quantify interocular suppression in intermittent exotropia (IXT) patients during the states of orthotropia and exodeviation.

**Methods:** The OKN motion in subjects (15 controls and 59 IXT subjects) who viewed dichoptic oppositely moving gratings with different contrast ratios was monitored and recorded by an eye tracker. Interocular suppression in control subjects was induced using neutral density (ND) filters. The OKN direction ratios were fitted to examine the changes of interocular suppression in subjects under different viewing states. Two established interocular suppression tests (phase and motion) were conducted for a comparative study.

**Results:** The dOKN test, which requires a minimal response from subjects, could accurately quantify the interocular suppression in both IXT and control subjects, which is in line with the established interocular suppression tests. Overall, although comparative, the strength of interocular suppression detected by the dOKN test (0.171 ± 0.088) was stronger than those of the phase (0.293 ± 0.081) and the motion tests (0.212 ± 0.068) in the control subjects with 1.5 ND filters. In IXT patients, when their eyes kept aligned, the dOKN test (0.58 ± 0.09) measured deeper visual suppression compared with the phase (0.73 ± 0.17) or the motion test (0.65 ± 0.14). Interestingly, strong interocular suppression (dOKN: 0.15 ± 0.12) was observed in IXT subjects during the periods of exodeviation, irrespective of their binocular visual function as measured by synoptophore.

**Conclusion:** The dOKN test provides efficient and objective quantification of interocular suppression in IXT, and demonstrates how it fluctuates under different eye positions.

## Introduction

Interocular suppression occurs during the dominance competition between two eyes and potentially contributes to the pathological processes of visual function impairment experienced by patients with amblyopia or strabismus (Maehara et al., 2011; Dorr et al., 2019; Webber et al., 2020). For strabismus, visual suppression has been proposed as the most important indicator for determining the intervention strategies in clinical practice (Kim et al., 2019; Yoo et al., 2019). However, the assessment of suppression in patients with intermittent exotropia (IXT) could be challenging because IXT patients sometimes are able to maintain ocular alignment and still have a certain degree of binocular visual function (Wakayama et al., 2013).

IXT is the most common type of divergent strabismus, occurring in more than half of patients with exotropia (Mohney and Huffaker, 2003; Bergholz and Salchow, 2015), and it has been well recognized as variable and unstable (Hatt et al., 2008; Bergholz and Salchow, 2015; Economides et al., 2016). These intrinsic characteristics of IXT make it difficult for eye care professionals to determine the ideal treatment strategy and intervention timing for patients. It is well recognized that surgical intervention decisions should be made more by the reference of a patient’s suppression depth rather than divergent degree alone, especially for children whose visual function is still developing. Normally, longer duration of deviation results in stronger visual suppression in patients with strabismus (Adams et al., 2017; Cadet et al., 2018). However, the situation is much more complicated in IXT patients, as their visual suppression condition is unstable and varies with eye position (Serrano-Pedraza et al., 2011; Wakayama et al., 2013). Patients with IXT can switch the fixating eye spontaneously according to the object position (Adams et al., 2017; Ramachandran and Das, 2020). Thus, suppression can change between eyes depending on fixation. Moreover, the deviated eyes are believed to be suppressed in the clinical practice. However, accumulating evidence shows that the foveal function of the deviated eye is not completely suppressed and still has perceptual functions (Economides et al., 2014, 2017; Agaoglu et al., 2015). Yet, visually dissimilar targets for the two eyes were used in these studies, which might cause fixation switch and result in incorrect measurement of suppression. Thus, an accurate objective quantification of visual suppression in patients with IXT is warranted.

Clinically, the most commonly used assessments for visual suppression are Bagolini glasses and Worth 4-dot tests. Though these tests are easy to operate, they are in fact subjective qualitative assessments to evaluate visual suppression from the foveal region of the fixating eye to the non-foveal region of the deviated eye in IXT patients when they exhibit exodeviation (Baker et al., 2008; Wen et al., 2018), thus the foveal-foveal suppression remains unknown. Meanwhile, electrophysiological (Brown et al., 1999; Zheng et al., 2019) and neuroimage (Lygo et al., 2021) methods as well as several established psychophysical paradigms, including the global motion coherence threshold, orientation coherence, and interocular phase combination (Narasimhan et al., 2012; Ding et al., 2013; Zhou et al., 2013; Wang et al., 2019; Zheng et al., 2019; Gong et al., 2020; Lygo et al., 2021), have been devised to quantify suppression. These subjective assessments require attention and certain responses from patients during evaluation, making them more difficult for young children. Moreover, in patients with strabismus, fixation switch would frequently occur because of the dichoptic stimulation (Barboni et al., 2020), and ocular position cannot be monitored in real time during these tests, thereby affecting the reliability of the results. Currently, there is no recognized objective quantification method for foveal-foveal visual suppression in IXT.

Optokinetic nystagmus (OKN) is an involuntary rhythmic ocular reaction elicited by moving patterns across the visual field (Lewis et al., 2000; Valmaggia et al., 2003). This visual field-motion response minimizes retinal slip and stabilizes retinal images, which can be quantified objectively and proposed as a good predictor for visual function. For example, it has been employed by eye care professionals to evaluate visual acuity in infants or applied for contrast function measurements (Hyon et al., 2010; Agaoglu et al., 2015; Wen et al., 2018; Doustkouhi et al., 2020). Also, during grating stimulations, subjects with interocular visual suppression exhibit asymmetric OKN movement, as the suppressed eye has difficulty in tracking moving gratings (Knapp et al., 2013; Agaoglu et al., 2015). Schor and Levi (1980) indicated that OKN motion was significantly decreased in the amblyopic eyes compared with the normal eyes, and the degree of decline was particularly related to the depth of visual suppression. Moreover, Wen et al. (2018) suggested that dichoptic OKN assessment could provide objective and reliable quantification of interocular suppression in patients with amblyopia. In OKN movement, there is a slow phase called pursuit in which the ocular moves toward the same direction as the stimulus, and a fast phase called saccade in which the ocular moves to the opposite direction (Knapp et al., 2013). During interocular rivalry, the OKN direction in the slow phase indicates the perceived direction of dominant motion, which is biased toward the stimulus with higher contrast (Knapp et al., 2013). Hence, by analyzing the OKN pattern, the dominance relationship between two eyes and the depth of interocular visual suppression could be calculated.

With the advent of an eye tracker using a desktop eye movement recorder, the dichoptic OKN motion can be measured and quantified objectively and easily and, more importantly, not restricted by the patient’s eye position. On this basis, in the present study, we sought to develop an objective quantification method for visual suppression in IXT subjects during the state of orthotropia and exodeviation, by assessing OKN behavior with an eye tracker. These observations might provide a new way to assess visual suppression and offer clinical guidance for patients with IXT.

## Materials and Methods

### Subjects

Fifty-nine IXT subjects (33 females and 26 males) and 15 control subjects (8 females and 7 males) were recruited for this study from Zhongshan Ophthalmic Center, Sun Yat-sen University. As shown in Table 1, the average subject age was 25.6 ± 4.29 years (range from 17 to 34 years, median age: 26 years) in healthy controls and 18.78 ± 6.2 years (range from 7 to 33 years, median age: 17 years) in IXT subjects. Of 59 IXT recruits 37 were children (62.7%), as IXT subjects tend to cease attending clinics by adulthood. This study was approved by the ethics committee of Sun Yat-sen University, Zhongshan Ophthalmic Center and was performed in compliance with the tenets of the Declaration of Helsinki. All enrolled subjects and their legal guardians voluntarily gave written informed consent before the experiments.

**TABLE 1:** Demographics of the control and IXT patients in the study.

	Control	IXT patients
Subjects	15	59
Sex (female/male)	8/7	33/26
Age (years)	25.60 ± 4.29	18.78 ± 6.2
Range	17–34	7–33
Median	26	17
Spherical equivalent (D)	2.56 ± 1.33	3.36 ± 1.54
LogMAR CDVA	–0.06 ± 0.05	0.01 ± 0.06
Strabismus angle (△)	–	35.45 ± 8.68
Stereoacuity (Titmus, arcsec)	20 ± 10	80 ± 15

### Eligibility

All IXT subjects were diagnosed by an experienced pediatric ophthalmologist (Prof. Deng). Standard ophthalmological examinations, including best-corrected visual acuity (BCVA), refractive error, ocular movements and alignment, fusion and stereopsis functions, slit lamp and fundus photography, were performed for all IXT and control subjects. The binocular single vision (BSV) of the IXT subjects was assessed and graded by synoptophore testing as first-degree with simultaneous perception, second-degree with sensory fusion, and third-degree with stereoscopic function. Depending on the BSV gradings, the IXT subjects were divided into two groups (Table 2). Subjects who accomplish all three grades of image slides were considered to possess all three levels of BSV and divided into the normal binocular function group (NBF). Otherwise, they were divided into the abnormal binocular function group (ABF).

**TABLE 2:** BSV grading in IXT subjects.

BSV	None	First-degree	Second-degree	Third-degree
	
	61%			39%
Numbers (Total: 59)	10 (17%)	8 (14%)	18 (30%)	23 (39%)

The inclusion criteria were as follows: (1) diagnosed with IXT, and shows exotropia since early childhood; (2) not suffering from amblyopia in either eye; (3) have equal refractive error with equivalent spherical differences less than 1 diopter (D) and astigmatism difference less than 0.5 D between the two eyes; (4) presented with no other ocular disease except IXT and refractive errors, and without any systemic diseases either; and (5) has the ability to alternate ocular fixation freely and has no sign of diplopia.

### Psychophysics Measurements for Visual Suppression

The phase, motion, and dOKN test were conducted for all subjects in the same dimly lit room (illumination of 100 lux). The visual stimuli were generated and controlled by MATLAB with Psychophysics Toolbox extensions (MathWorks, Inc., Natick, MA) displayed on an ASUS 3D monitor (VG278, 144-Hz refresh rate, 1,920*1,080 resolution, Taiwan). The subjects’ heads were fixed with the help of a chin rest, and the viewing distance to the monitor (597 mm*336 mm, background brightness of 35 cd/m^2^) was 57 cm. During stimulation, NVIDIA 3D VISION LCD shutter glasses (Santa Clara, CA) were used to create a dichoptic viewing state on the subjects. An extra pair of refractive-corrected glasses were used in the subjects with refractive errors.

#### Phase Test

The phase tests were performed according to a previous study (Zhou et al., 2013; Chen et al., 2016, 2018). Briefly, a fixation cross with four complementary dots was presented to assist fusion for the subjects under a dichoptic viewing state (Ge et al., 2020). Then, two horizontal sinusoidal gratings (size: 5.7°*5.7°) with 45° offset phase difference in orientation, which contain 2 cycles at a spatial frequency of 0.293 cpd, were presented dichoptically. The grating contrast in the non-dominant eye was fixed at 100%. Subjects were required to judge the perceived phase. The phase difference was measured when the interocular contrast ratios were at 0, 0.1, 0.2, 0.4, and 1. All tests were repeated at least 4 times. The obtained data was fitted using a Curve Fitting toolbox incorporated in MATLAB (Chen et al., 2016). The equation for data analysis is presented as follows. φ represents the measured perceived phase; δ represents the interocular contrast ratio; α represents the effective contrast ratio (ECR) when the binocular phase perception reaches a balance point; r represents a non-linear factor; θ represents the intraocular phase difference, which is 45° in the present study.


$$\varphi =2\,\tan^{-1}\left[\frac{1-{\left(\delta /\alpha \right)}^{1\,r}}{1\,{\left(\delta /\alpha \right)}^{1\,r}}\cdot \tan\left(\frac{\theta }{2}\right)\right]$$

#### Motion Test

For a motion test (Chen et al., 2016), the fixation tasks were conducted as the phase test. Afterward, visual signals and noise dots were constantly presented at a speed of 2°/s for both eyes to obtain a motion coherence ratio. Then, the signal and noise dots were presented to the dominant and the non-dominant eye at their fixed motion coherence ratio, respectively. The contrast of the noise dots was varied, and the contrast of signal dots was fixed at 100%. The effective contrast ratio (ECR) of motion test was defined as the ratio between the contrast of dominant and non-dominant eye. In each frame, a maximum of 50 dots inside a circular aperture (diameter = 11.4°) was presented. The size of dots was randomized in a range of ± 20% (average: 1.1°).

#### Dichoptic Optokinetic Nystagmus Test

**FIGURE 1:**
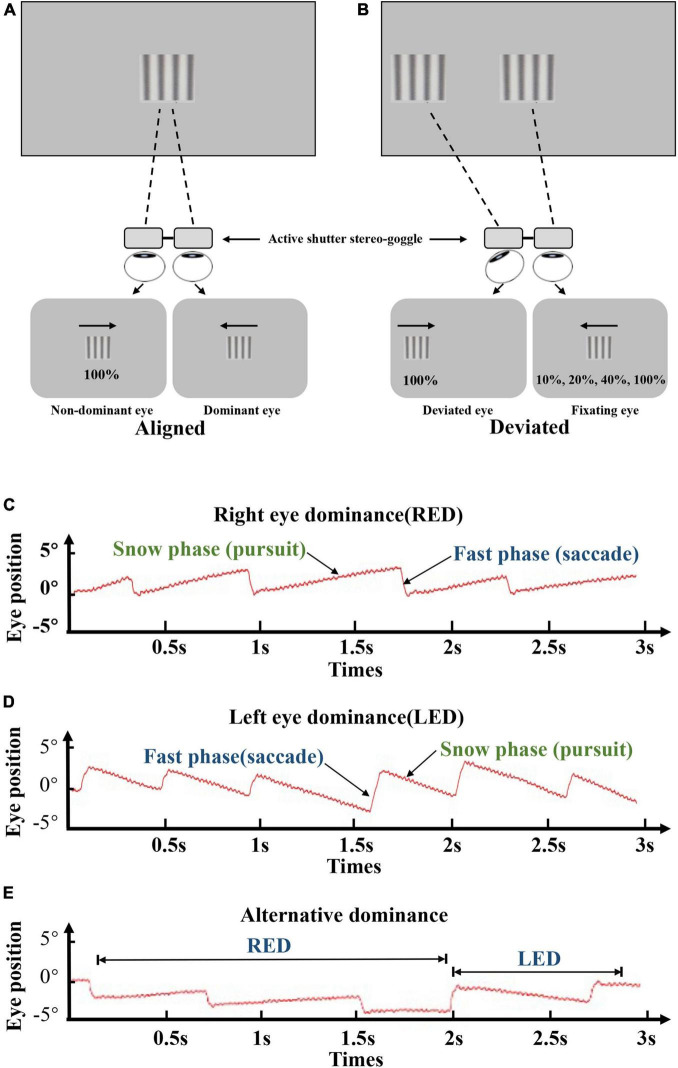
Visual suppression by dOKN test. **(A,B)** All subjects were instructed to track two opposite drifting gratings at the distance of 57 cm, under the dichoptic state by using 3D VISION LCD shutter glasses. Both eyes were stimulated with different visual inputs: the non-dominant/deviated eyes were treated with high contrast grating (100%), and the dominant eyes were treated with varied contrast grating (10, 20, 40, and 100%). For healthy controls, interocular suppression was induced by placing neutral density (ND) filters (0, 0.9, or 1.5 ND) in front of their non-dominant eye. All stimulations lasted for 3 s followed by a 2-s break and were repeated 15 times. **(C–E)** Red lines represent the ocular moving path during OKN stimulation. The moving pattern of the slow phase suggests the dominant eye. Right eye dominance **(C)**, left eye dominance **(D)**, and alternating dominance **(E)**.

For the dOKN test, the subjects were instructed to track two repetitive oppositely moving gratings under dichoptic conditions with 3D VISION LCD shutter glasses. As shown in Figure 1, the quadrate black and white sinusoidal grating visual mark (size: 10°*10°) was presented to each eye on the display screen according to the current eye position that was measured by the eye tracker. The gratings were moving in an oriented direction, from temporal side to nasal side, at the same velocity (10°/s) with a spatial frequency of 3 cpd. The right eyes were stimulated by gratings that moved from right to left, and the left eyes were stimulated by gratings that moved from left to right. Each stimulation lasted for 3 s followed by a 2-s break and were repeated 15 times for each contrast ratio, followed by a 1-min rest period. During each break time, a fixation target was given to the dominant eye, so that the fixation eye would not shift and continue to be the dominate eye. Different visual inputs were used: the non-dominant/deviated eyes were treated with high contrast grating (100%), and the dominant/fixating eyes were treated with varied contrast grating (10, 20, 40, and 100%, Figure 1A). During the test, the ocular movement was monitored and recorded continuously and intuitively by an eye tracker (Eye Link 1000 Tower; SR Research), with a sampling rate of 1,000 Hz. If their eye position was found to be not well controlled as instructed, they would be reminded promptly to concentrate.

In healthy controls, the eye dominance was determined by card-hole method. The dOKN test was conducted in a state that interocular suppression was induced with neutral density (ND) filters (Kodak Wratten; Eastman Kodak, Rochester, NY; 0.3-log unit increments) placed in front of their non-dominant eye. Three ND filters with different intensities were used: 0, 0.9, or 1.5 ND. No light is reduced by 0 ND; only 1/8 amount of light can go through a 0.9 ND filter; and only 1/32 amount of light can go through a 1.5 ND filter. In IXT subjects, the dOKN test was conducted under the state of orthotropia and exodeviation, respectively. Briefly, the main deviated eye and dominant eye were determined by the cover and uncover test and the card-hole method (Yang and Hwang, 2011), and exotropia was induced by covering the eye for 30 min. Then, their deviation angle was measured by alternating prism cover test. The initial grating position on the screen was adjusted in real-time (Figure 1B) according to the ocular deviation recorded by the eye tracker, to ensure foveal presentation in the two eyes. The main-deviated eyes were stimulated with high contrast grating (100%), and the fixating eyes were treated with varied contrast grating (10, 20, 40, and 100%, Figure 1B) randomly. All stimulations lasted for 3 s followed by a 2-s break and were repeated 15 times.

### Analysis for Dichoptic Optokinetic Nystagmus Test

In the present study, the OKN pursuits were visualized and analyzed by SR Research, an eye tracking software program. The dOKN data when subjects were blinking or unfocused would be discarded. The validity of whether the OKN recording could reflect objective motion perception was confirmed in healthy controls, who were instructed to view moving stimuli (data not shown), which is consistent with a previous study (Wen et al., 2018).

#### Optokinetic Nystagmus Directional Ratio and Intraocular Contrast Ratio

By analyzing the contrast ratio of dichoptic gratings when the OKN distribution is balanced in both eyes, the depth of visual suppression can be calculated and quantified. Briefly, when the eyes moved leftward in the slow phase and moved rightward in the fast phase, OKN pursuits were induced by the stimulation of the right eye, indicating that the right eye dominated at that moment, and vice versa. The OKN directional ratio is defined as the difference in dominance times between two eyes divided by their sum (equation as follows). Its value ranges from –1 to 1, while –1 indicates dominant/fixating eye preference, and 1 indicate non-dominant/deviated eye preference. The intraocular contrast ratio is defined as the difference in contrast of gratings presented for both eyes (equation as follows).

For healthy controls:


OKNdirectionalratio=(non-dominantOKN-dominantOKN)/(non-dominantOKN+dominantOKN)

intraocular⁢contrast⁢ratio=grating⁢contrast⁢of  dominant⁢eye/grating⁢contrast⁢of⁢non-dominant⁢eye.

For IXT subjects:


OKNdirectionalratio=(deviatedOKN-fixatingOKN)/(deviated⁢OKN+fixating⁢OKN)

intraocular⁢contrast⁢ratio=grating⁢contrast⁢of  fixating⁢eye/grating⁢contrast⁢of⁢deviated⁢eye.

#### Fitting Curve

**FIGURE 2:**
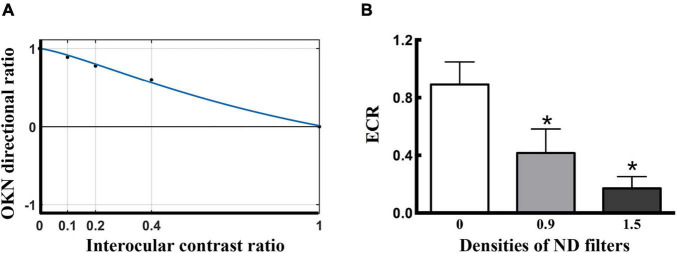
Visual suppression measured by dOKN test in control subjects. **(A)** The fitting curve of a healthy control. The *x*-axis represents the grating contrast ratio (dominant/non-dominant), and the *y*-axis represents the OKN directional ratio. In normal subjects, the suppression depth monotonically decreased with increasing stimuli contrast to the fellow eyes. The ECR is defined as the contrast ratio of the interocular balance points, which is the corresponding value of the *x*-axis when *y* = 0 that both eyes dominate in a balanced manner. **(B)** The ND filter induced visual suppression in the healthy controls. *Represented statistical difference.

To quantify the visual suppression, the data obtained from the dOKN test was fitted using a Curve Fitting toolbox, as the phase test. The *x*-axis represents the intraocular contrast ratio, and the *y*-axis represents the OKN directional ratio (Figure 2A, a healthy control). A similar non-linear binocular combination model as used in the phase test was adopted to fit the OKN performance. δ represents the intraocular contrast ratio; φ represents OKN directional ratio; α represents ECR; and *r* represents non-linear factor.


$$\varphi =8\,\text{atan}\,\left(0.4142^{*}\,\frac{\alpha^{1\,r}-\delta^{1\,r}}{\alpha^{1\,r}+\delta^{1\,r}}\right)\cdot \frac{1}{\pi }$$

#### Effective Contrast Ratio

When the OKN directional ratio equals 0 (*y* = 0), the interocular rivalry reaches a balance point in the subjects, and its corresponding *x*-axis value (intraocular contrast ratio) is defined as ECR, which reflects the interocular visual suppression. ECR value ranges from 0 to 1, 0 indicates complete intraocular suppression in the non-dominant eye, while 1 indicates balanced interocular rivalry.

### Statistical Methods

The clinical data are presented as the mean ± standard deviation (SD), and normality was tested by the Shapiro–Wilk test. Data that are normally distributed were analyzed with paired *t*-tests and ANOVA; otherwise, they were analyzed with non-parametric statistics. The ECR evaluated by different methods was compared by using Kruskal-Wallis H-tests. The correlation between the dOKN test and other methods was measured with Spearman’s correlation. The correlation between ECR and the interocular vision function was analyzed by the chi-square test. All statistical analyses were performed with SPSS Statistics 23 (SPSS Inc., Chicago, IL). A *p*-value less than 0.05 was considered statistically significant.

## Results

### Dichoptic Optokinetic Nystagmus Test for the Quantification of Visual Suppression

Visual suppression is a contrast-dependent phenomenon, which reaches its maximum when the dominant eye views high-contrast stimuli (de Graaf et al., 2017). During the dOKN test, the subjects were stimulated by repetitive, oppositely moving visual gratings with different contrast ratios, under the dichoptic viewing state, as we described in “Materials and Methods” section (Figures 1A,B). The stimulated OKN motion pattern was recorded by an eye tracker.

The OKN direction is related to the direction of moving stimulus. As shown in Figures 1C–E, the red lines represent the OKN moving path: ocular tracking the gratings in the slow phase (pursuit), and moving back to the fixation point in the fast phase (saccade). The moving pattern in the slow phase indicates which is the dominant eye. Figure 1C presents the OKN moving path of a subject whose right eye shows dominance (RED), and Figure 1D belongs to a subject whose left eye is the dominant eye (LED). When subjects’ eyes change dominance alternatively, their OKN motion pattern appears as shown in Figure 1E.

### Quantification of Visual Suppression in Control Subjects by the Dichoptic Optokinetic Nystagmus Test

To evaluate the efficacy of the dOKN test in visual suppression assessment, 15 healthy controls were included and tested as described previously in “Materials and Methods” section. The depth of suppression in the non-dominant eye was modulated with different ND filters. The closer the fitted ECR was to 0, the deeper the subjects’ visual suppression. The response from a healthy control subject is shown in Figure 2A, whose ECR = 1, namely, OKN distribution reached a balance point (*y* = 0) when the interocular contrast ratio is equal to 1 (*x* = 1), suggesting a balanced binocular perception of the moving gratings. The dOKN results showed that the OKN ECR was 0.891 ± 0.156 in control subjects with no ND filter (Figure 2B), suggesting relatively balanced binocular perception and weak visual suppression. While, by using ND filters with different densities, various degrees of suppression were induced in the healthy controls and a linear correlation was found (0 ND: 0.891 ± 0.156; 0.9 ND: 0.428 ± 0.197; and 1.5 ND: 0.177 ± 0.102, *F* = 3.987, *p < 0.05, one-way analysis). The test-retest results suggested good repeatability in dOKN test. Spearman’s correlation analysis revealed a positive correlation between the second test and first test results of the dOKN ECR (*r* = 0.768, *p < 0.05).

### The Dichoptic Optokinetic Nystagmus Test Is Comparable With the Established Visual Suppression Measurements

**FIGURE 3:**
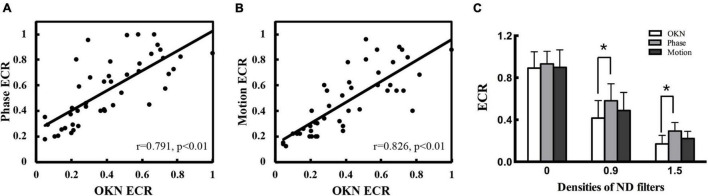
Comparison of the dOKN test with phase and motion tests in control subjects. **(A)** The visual suppression results of the dOKN test were positively correlated with that of the phase test. **(B)** The visual suppression results of the dOKN test were positively correlated with that of the motion test. **(C)** The visual suppression measured the dOKN test as well as the phase and motion tests in normal subjects under various ND filters. Deep visual suppression was induced in normal subjects under the 1.5 D filter. *Represented statistical difference.

In addition, we compared the ECR values of the dOKN test with those of established visual suppression measurements, the phase and the motion tests, in healthy controls using ND filters. The Spearman correlation analysis results showed that the dOKN ECRs were positively correlated with the results of either phase test (*r* = 0.791, *p < 0.05, Figure 3A) or motion test (*r* = 0.826, *p < 0.05, Figure 3B).

Moreover, the results obtained from these three tests exhibited similar tendency in healthy controls, showing deep visual suppression under the 1.5 D filter and weak suppression with no filter (Figure 3C). However, differences between these three tests were still observed. First, the results obtained from the dOKN test under the 1.5 ND filter (0.171 ± 0.088) were quite close to those of the motion test (0.212 ± 0.068, *p* > 0.05), but exhibited a significant difference from those of the phase test (0.293 ± 0.081, *p < 0.05, LSD comparison). The same phenomena were observed when the subjects were under the 0.9 ND filter (OKN: 0.416 ± 0.167, phase: 0.581 ± 0.162, and motion: 0.488 ± 0.172, *p < 0.05). Second, the visual suppression depth measured by the dOKN test is generally deeper than those of the phase and the motion test. Third, the visual suppression results measured by the phase and the motion tests were not significantly different, which is consistent with previous reports.

**FIGURE 4:**
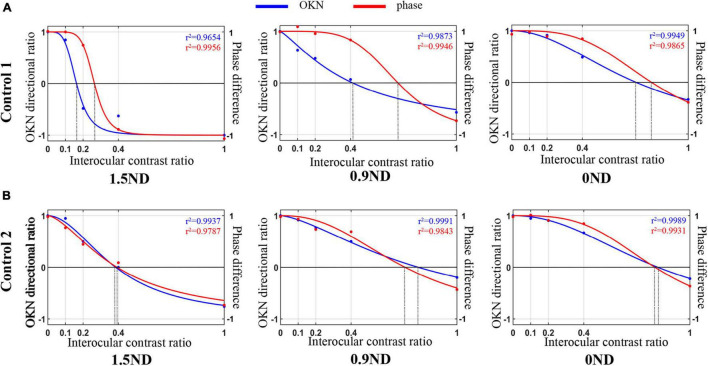
Comparison of the dOKN test and phase tests in control subjects. **(A)** Control 1. **(B)** Control 2. The fitting curves of two healthy controls’ curve fittings obtained from the dOKN test (blue line) and the phase method (red line). The *x*-axis represents the grating contrast ratio (dominant/non-dominant), and the *y*-axis represents the OKN directional ratio or the phase difference.

The data of the dOKN test (blue line) and the phase test (red line) obtained from two healthy controls were plotted in Figure 4. Generally, the variation trend of the visual suppression in the subjects with different interocular contrast ratios obtained from the dOKN test is similar to that of the phase test. In some subjects (Control 1), the OKN and phase distribution showed a high degree of coincidence; however, in some subjects they did not (Control 2).

### Visual Suppression Quantified by Dichoptic Optokinetic Nystagmus Test in Intermittent Exotropia Subjects During the States of Orthotropia and Exodeviation, Respectively

**FIGURE 5:**
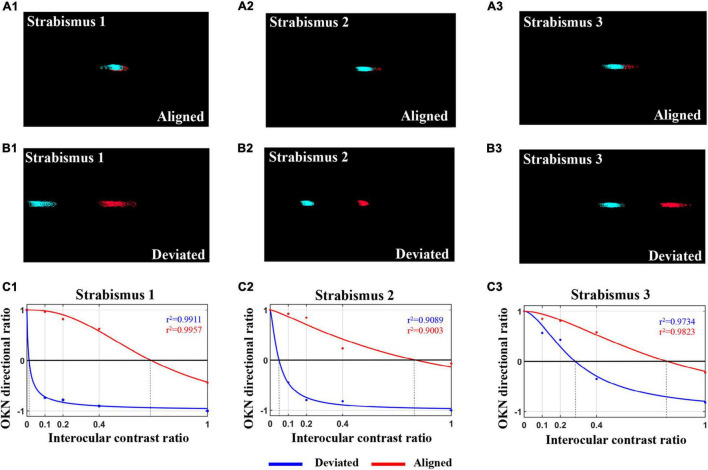
The OKN distribution in IXT subjects when kept alignment shows deviation. **(A1–3)** The eye movement pattern of three IXT subjects when kept alignment during the dOKN test, as recorded by an eye tracker. **(B1–3)** The eye movement pattern of three IXT subjects when showing deviation during the dOKN test, as recorded by an eye tracker. **(C1–3)** The fitting curves drawn by dOKN of three IXT subjects when kept alignment (red line) or manifesting exotropia (blue line).

Interocular suppression was assessed in 59 IXT subjects under the states of orthotropia and exodeviation. As mentioned in “Materials and Methods” section, an eye tracker was employed to monitor the subjects’ eye positions and record ocular movement patterns in real time during dOKN test. As shown in Figures 5A1–3, the ocular motion trace of both eyes is mostly overlapped, indicating that the deviation angle was less than 5°and the subjects were maintaining alignment during the recording time. While, when the ocular motion trace of both eyes separated by a certain distance (Figures 5B1–3), indicating that deviation manifest in the subjects.

Figures 5C1–3 presents the fitting curve of three IXT subjects when deviation manifested (blue line) or maintained alignment (red line). Our data showed that, in the IXT subjects who exhibited deviation, the deviated eyes were almost completely suppressed when the fixating eyes were stimulated with high contrast gratings, while the deviated eyes could still track the gratings when low contrast stimuli were shown to the fixating eyes.

When IXT subjects maintained binocular alignment (Figures 5A1–3), the dOKN, phase, and motion tests were performed. During the examinations, the subjects were monitored and constantly instructed to concentrate and maintain eye alignment. At this point, the interocular visual suppression measured by the dOKN test was 0.58 ± 0.09, showing significantly stronger suppression compared with healthy controls (0.891 ± 0.156) under no filter (*p < 0.05). However, neither the phase (0.73 ± 0.17) nor the motion test (0.65 ± 0.14) reached statistical significance between IXT subjects when aligned and healthy controls (*p* > 0.05). Moreover, as shown in Table 3, the suppression depth measured by the dOKN (0.58 ± 0.09) was smaller than that of both the phase (0.73 ± 0.17) and motion tests (0.65 ± 0.14; *p < 0.05), suggesting deeper visual suppression, which is consistent with the results obtained from healthy controls.

**TABLE 3:** The ECR of IXT.

Eye position	dOKN	Phase	Motion
Alignment	0.58 ± 0.09	0.73 ± 0.17	0.65 ± 0.14
Misalignment	0.15 ± 0.12		

When deviation showed in the IXT subjects, the depth of visual suppression was quantified by dOKN test (Figures 5B1–3), during which the stimulating gratings were presented on the screen according to the subjects’ eye positions (Figure 1). Once deviation was manifested in IXT subjects, deep interocular suppression appeared (ECR aligned: 0.58 ± 0.09, ECR deviated: 0.15 ± 0.12, *p < 0.05, Table 3). Moreover, their interocular suppression increased with increasing contrast of the stimulation for the fixating eyes.

**FIGURE 6:**
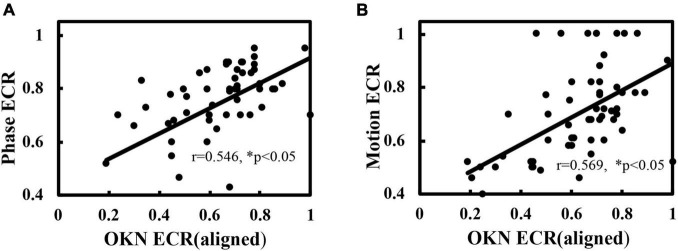
Correlation of the dOKN test with phase and motion tests in IXT subjects when aligned. **(A)** The visual suppression results of the dOKN test were positively correlated with that of the phase test. **(B)** The visual suppression results of the dOKN test were positively correlated with that of the motion test. *Represented statistical correlation.

In addition, consistent with the analysis of healthy controls, positive correlations between the dOKN test and established tests (phase and motion) were observed in IXT subjects when correct binocular alignment was maintained (phase vs. dOKN: *r* = 0.546; motion vs. dOKN: *r* = 0.569, *p < 0.05, Figures 6A,B), further confirming the reliability of the dOKN test in visual suppression quantification. However, differences between these tests were still observed. The depth of visual suppression measured by the dOKN test was generally deeper than that of the phase and the motion tests.

### Visual Suppression in Intermittent Exotropia Subjects With Normal or Abnormal Binocular Visual Function

**FIGURE 7:**
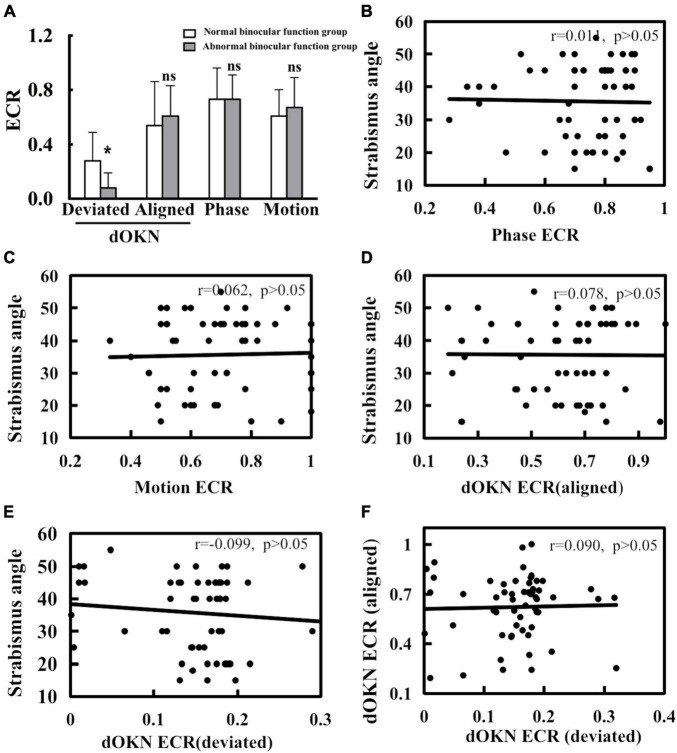
Correlation of visual suppression and strabismus angle. **(A)** When deviation was shown, a significant difference in dOKN results was observed between the normal binocular function (NBF) group and the abnormal binocular function (ABF) group. **(B)** No significant correlation exists between deviation angle and visual suppression measured by phase test. **(C)** No significant correlation exists between deviation angle and visual suppression measured by motion test. **(D)** No significant correlation exists between deviation angle and visual suppression measured by the dOKN test when kept aligned. **(E)** No significant correlation exists between deviation angle and visual suppression measured by the dOKN test when deviation shows. **(F)** No significant correlation exists in between the dOKN ECR measured under alignment state with that under the deviation state. *Represents statistical difference. Neither phase nor motion test could reflect this discrepancy.

The BSV of the IXT subjects was assessed by synoptophore test. Depending on the BSV gradings, the IXT subjects were divided into two groups (Table 2). The IXT subjects who accomplish all three degrees of image slides were considered to possess all three levels of BSV and were divided into the normal binocular function group (NBF) (23, 39%). Otherwise, they were divided into the abnormal binocular function group (ABF) (36, 61%). As shown in Figure 7A, when subjects manifested deviation, deep interocular suppression was reflected by the dOKN test, in both the NBF group (dOKN-d: 0.28 ± 0.21) and the ABF group (dOKN-d: 0.08 ± 0.11). While, when subjects were kept aligned, relatively weak suppression was measured by the dOKN test, phase, and motion tests (dOKN-a, NBF group: 0.54 ± 0.33, ABF group: 0.61 ± 0.22; phase, NBF group: 0.73 ± 0.18, ABF group: 0.73 ± 0.23; motion, NBF group: 0.61 ± 0.19, ABF group: 0.67 ± 0.22, *p* > 0.05).

Moreover, a significant difference in dOKN results was observed between the NBF group and the ABF group when deviation was manifested (*p < 0.05), showing various visual suppression depth. However, when subjects maintain alignment, no significant difference was observed in the dOKN results between these two groups, nor was any difference observed in the phase and the motion test (*p* > 0.05).

In addition, in order to explore the relationship between patients’ strabismus condition and their visual suppression, we further analyzed the correlation between the deviation angle magnitude and visual suppression in IXT subjects. Our data showed no significant correlation in the phase or the motion test (for phase: *r* = 0.011; for motion: *r* = 0.062; *p* > 0.05, Figures 7B,C). Consistently, no significant correlation was observed between the deviation angle magnitude and visual suppression measured by dOKN test either (dOKN-a: *r* = 0.078; dOKN-d: *r* = 0.099; *p* > 0.05, Figures 7D,E). Furthermore, there was also no correlation between the dOKN test results of the IXT subjects with different eye position (*r* = 0.09, *p* > 0.05, Figure 7F), which is in accordance with the variable characteristics of IXT patients.

## Discussion

Visual suppression has been proposed as an important anti-diplopia mechanism in patients with IXT (Serrano-Pedraza et al., 2011; Adams et al., 2017). Whether for clinical diagnosis, intervention strategy guidance, or postoperative visual function evaluation, the measurement of visual suppression is of great clinical importance. It helps to determine the surgical intervention timing, assess postoperative visual function re-establishment, etc. However, there is no clinically accepted objective quantitative assessment of visual suppression, especially in patients with strabismus. In the present study, our data suggest that, with the assistance of eye trackers, the dOKN test can objectively quantify the changes in intraocular suppression not only in healthy controls but also in IXT patients under various viewing conditions, which effectively complement the conventional suppression tests and provide intuitive evaluation to ophthalmologists and optometrists.

In healthy controls with ND filters, the visual suppression measured by the dOKN test showed a good concordance with those of the phase and the motion tests. Also, the variation trend of interocular suppression in healthy subjects induced by different ND filters could be reflected by the dOKN test accurately. Interestingly, we noticed that the dOKN test measured the smallest ECR value, thus showing the deepest visual suppression among the three tests. Several reasons may account for this observation. First, there were different cortical areas responsible for these three tests. The phase test was thought to reflect the function of the primary visual cortex V1, while the motion and the OKN tests (Lewis et al., 2000) might indicate the function of the middle temporal V5 region (Hess et al., 2014). Second, the inspection processes are quite different. For example, there is no time limit for the phase test, thus, subjects can allocate attention continuously and choose to proceed by themselves, while the dOKN and motion tests require subjects to make judgments within a short time. Third, the difference in inspection principles may also lead to different detection accuracies. The suppression mechanisms might differ between dynamic and static images, for instance. In addition, we could not rule out the possibility that different methodologies could have confounded the interpretation of our results.

As described above, for IXT patients with aligned eye position, dOKN, phase, and motion tests could complement one another. However, for IXT patients with large angle of ocular deviation, neither phase nor motion test is easy to perform, as the ocular deviation angle needs to be compensated, and the bi-foveal presentation of stimuli cannot be guaranteed because of the lack of continuous eye position monitoring. Besides, both the phase and the motion tests require attention and response from subjects, making these psychophysical tests difficult to carry out in young children. In contrast, the dOKN test is available and relatively easy to perform, regardless of the viewing state of subjects. More importantly, this paradigm is designed based on an involuntary ocular motion that requires a minimal response from subjects, making it more applicable during clinical practice. These observations are consistent with a previous study (Wen et al., 2018).

In patients with strabismus, interocular suppression is elicited to avoid diplopia (de Graaf et al., 2017). Consequently, the retina region that corresponds to the overlapping areas is suppressed. Though the foveal region in the deviated eye was once believed to be suppressed (Pratt-Johnson and Tillson, 1984), recent studies have revealed a different scenario. Economides et al. (2012) found that regardless of which eye is fixed, the foveal and temporal visual field in the deviated eye could still perceive visual signals. Agaoglu et al. (2015) also showed that in macaques with exotropia, OKN could be induced even when the stimulus pattern was presented only in the deviation visual field. Consistently, in the present study, typical OKN motion was induced in both eyes of IXT subjects regardless of the current eye position, as long as the visual stimulation was strong enough. When the moving visual stimuli were strong for the deviated eye and weak for the dominant eye, the deviated eye of IXT subjects could functionally respond to it, further indicating that the foveal region of the deviated eye is not totally suppressed. Generally, the results of the dOKN test follows the same general properties as other suppression measurement tests, but we further confirm that visual suppression is not binary. Not only that, we also observed much stronger visual suppression in the foveal region of the deviated eye in the IXT subjects during the state of exodeviation, compared with the state of normal alignment. Our results support the clinical knowledge that suppression can change with ocular alignment, and that alignment state needs to be considered when measuring suppression.

Moreover, the results of dOKN are in line with the categorization of binocular function measured by synoptophore, and this correlation was not observed in either the phase or the motion test (Figure 7A), further suggesting the potential clinical significance of the dOKN test. On one hand, this observation is consistent with the variability nature of IXT (Wakayama et al., 2013). On the other hand, the difference of intraocular suppression between the two subgroups was only evident when their deviation was manifested, thus the phase and motion tests might fail to capture this feature. Additionally, the depth of suppression measured with dOKN did not show any correlation with the deviation angle, and neither did the phase nor the motion tests. This evidence indicates that the deviation angle might not be a good indicator of binocular function in patients with IXT. Clinically, it is not rare to see the patients with small deviation degree have poor vision function. These patients need surgical intervention, by which to restore or maintain their visual function (Willshaw and Keenan, 1991). Thus, quantify interocular suppression in IXT patients objectively might help in the clinical decision of optical or surgical intervention for IXT patients in the future. Yet, further investigation is warranted.

This study had several limitations. First, as most adult IXT patients do not wish to receive clinical intervention, most IXT subjects in the present study were teenagers who needed surgical intervention, which might have caused selection bias. Second, the sample size was relatively small. The correlation between deviation angle and the depth of visual suppression was not observed. Third, though the dOKN test is easy to implement, it still needs subjects to focus and trace the grating targets as instructed, which is not suitable for young infants and patients with cognitive dysfunction. Hence, the clinical application of the dOKN test requires further investigation.

## Conclusion

In conclusion, our study shows that, with the assistance of an eye tracker, the dOKN test allows the objective and effective quantification of visual suppression for IXT patients, regardless of during the periods of orthotropia or exotropia. This test could not only complement the conventional suppression tests and provide a more comprehensive evaluation for strabismus patients but also provide an approach to explore the disease progression of IXT from another perspective.

## Data Availability Statement

The raw data supporting the conclusions of this article will be made available by the authors, without undue reservation.

## Ethics Statement

The studies involving human participants were reviewed and approved by the Ethics Committee of Sun Yat-sen University, Zhongshan Ophthalmic Center. Written informed consent to participate in this study was provided by the participants’ legal guardian/next of kin.

## Author Contributions

XC, ZC, YL, and DD collected and analyzed the data. XC, DD, and MY interpreted the data. XC, ZC, and MY were the major contributors in writing the manuscript. All authors read and approved the final manuscript.

## Conflict of Interest

The authors declare that the research was conducted in the absence of any commercial or financial relationships that could be construed as a potential conflict of interest.

## Publisher’s Note

All claims expressed in this article are solely those of the authors and do not necessarily represent those of their affiliated organizations, or those of the publisher, the editors and the reviewers. Any product that may be evaluated in this article, or claim that may be made by its manufacturer, is not guaranteed or endorsed by the publisher.

## Supplementary Material

Supplementary Figure 1 Visual suppression measured by psychophysics measurements. **(A)** Visual suppression measured by phase test. Subjects were instructed to view two horizontal sinusoidal gratings under dichoptic viewing state with the help of 3D shutter glasses. **(B)** Visual suppression measured by motion test. The signal and noise dots were presented to the dominant and the non-dominant eye at their fixed motion coherence ratio, dichoptically. For both phase and motion test, the contrast ratio of the grating for the non-dominant eye was fixed at 100%, while it varied for the dominant eye.
