# Supplementary figures and images for: A Dichoptic Optokinetic Nystagmus Paradigm for Interocular Suppression Quantification in Intermittent Exotropia
Source: Front Neurosci. 2021 Dec 3;15:772341. doi: 10.3389/fnins.2021.772341 (PMC8678071; doi:10.3389/fnins.2021.772341)

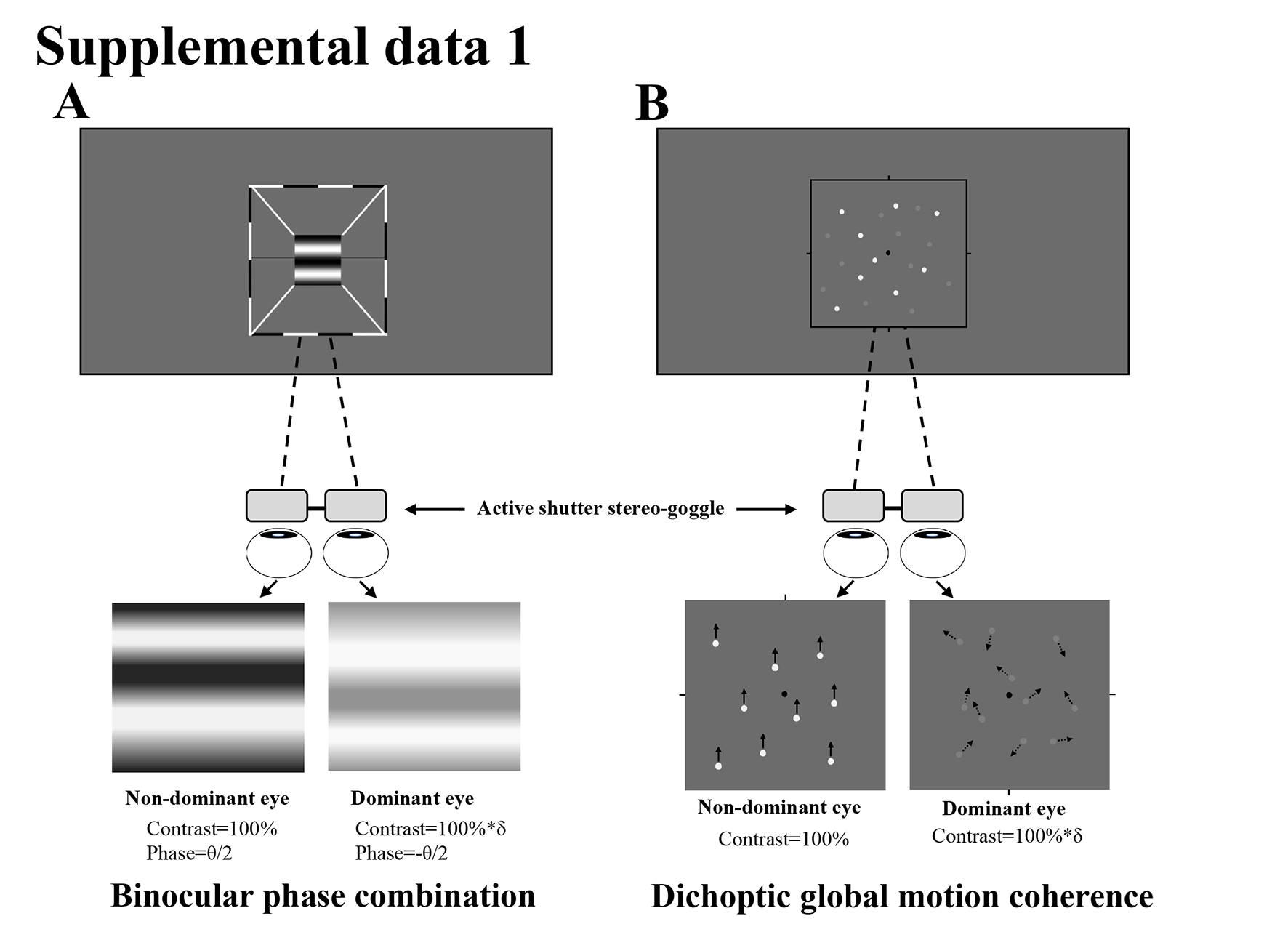

Supplement: Supplementary Figure 1 — Visual suppression measured by psychophysics measurements. (A) Visual suppression measured by phase test. Subjects were instructed to view two horizontal sinusoidal gratings under dichoptic viewing state with the help of 3D shutter glasses. (B) Visual suppression measured by motion test. The signal and noise dots were presented to the dominant and the non-dominant eye at their fixed motion coherence ratio, dichoptically. For both phase and motion test, the contrast ratio of the grating for the non-dominant eye was fixed at 100%, while it varied for the dominant eye. [file Image_1.TIF]
